# Supplementary material for: Dissipation of electron-beam-driven plasma wakes
Source: Nat Commun. 2020 Sep 21;11:4753. doi: 10.1038/s41467-020-18490-w (PMC7506535; doi:10.1038/s41467-020-18490-w)
Supplement: Supplementary file 1 — Supplementary Information [file 41467_2020_18490_MOESM1_ESM.pdf]

Supplementary Information for

Dissipation of electron-beam-driven plasma wakes

Rafal Zgadzaj, T. Silva, V. K. Khudyakov, A. Sosedkin, J. Allen, S. Gessner, Zhengyan Li, M. Litos, J. Vieira, K. V. Lotov, M. J. Hogan, V. Yakimenko, & M. C. Downer *

* Corresponding author. E-mail: downer@physics.utexas.edu

The PDF file includes:

Supplementary Figures **1**

Supplementary Figure 1: Lithium density profile in heat-pipe oven. 1

Supplementary Figure 2: Energy spectrum of spent SLAC bunch. 2

Supplementary Figure 3: Effect of multi-shot averaging on probe data. 3

Supplementary Figure 4: Trajectories of probe pulse rays through plasma column. 4

Supplementary Figure 5: Deceleration rate of fast electrons in medium of Maxwellian electrons. 5

Supplementary Figure 6: Contributions of various Li species to refractive index of e-beam-excited plasma column. 6

Supplementary Methods **7**

Longitudinal lithium vapor density profile along lithium heat-pipe oven 7

E-beam energy deposition into, and head erosion in, Li plasma 7

Single-shot vs. 30-shot-averaged probe images 8

Probe ray tracing for different incidence angles 9

Test of collisional scheme in LCODE 10

Atomic, ionic and plasma contributions to Li refractive index 11

Supplementary References **12**

Supplementary Figures

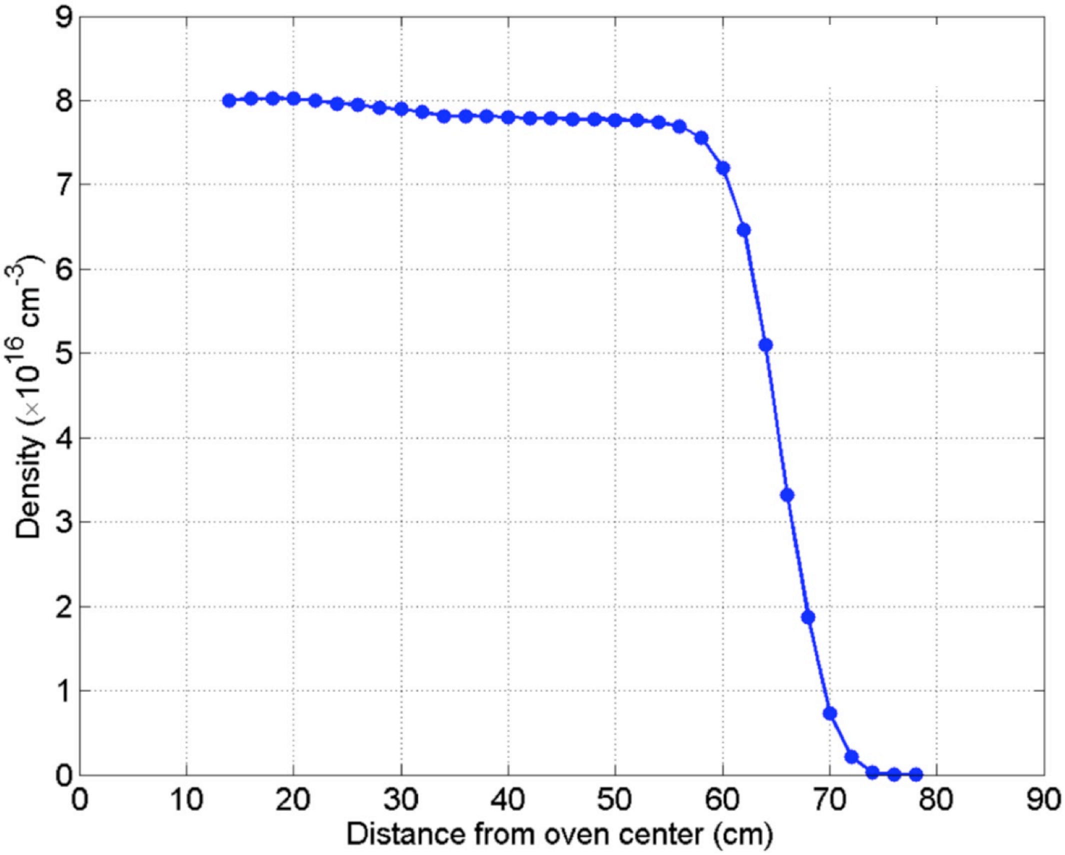

Supplementary Figure 1: Lithium density profile in heat-pipe oven.

Atomic density, determined from temperature measurements along oven axis, is plotted from 12 cm downstream of oven center to oven exit.

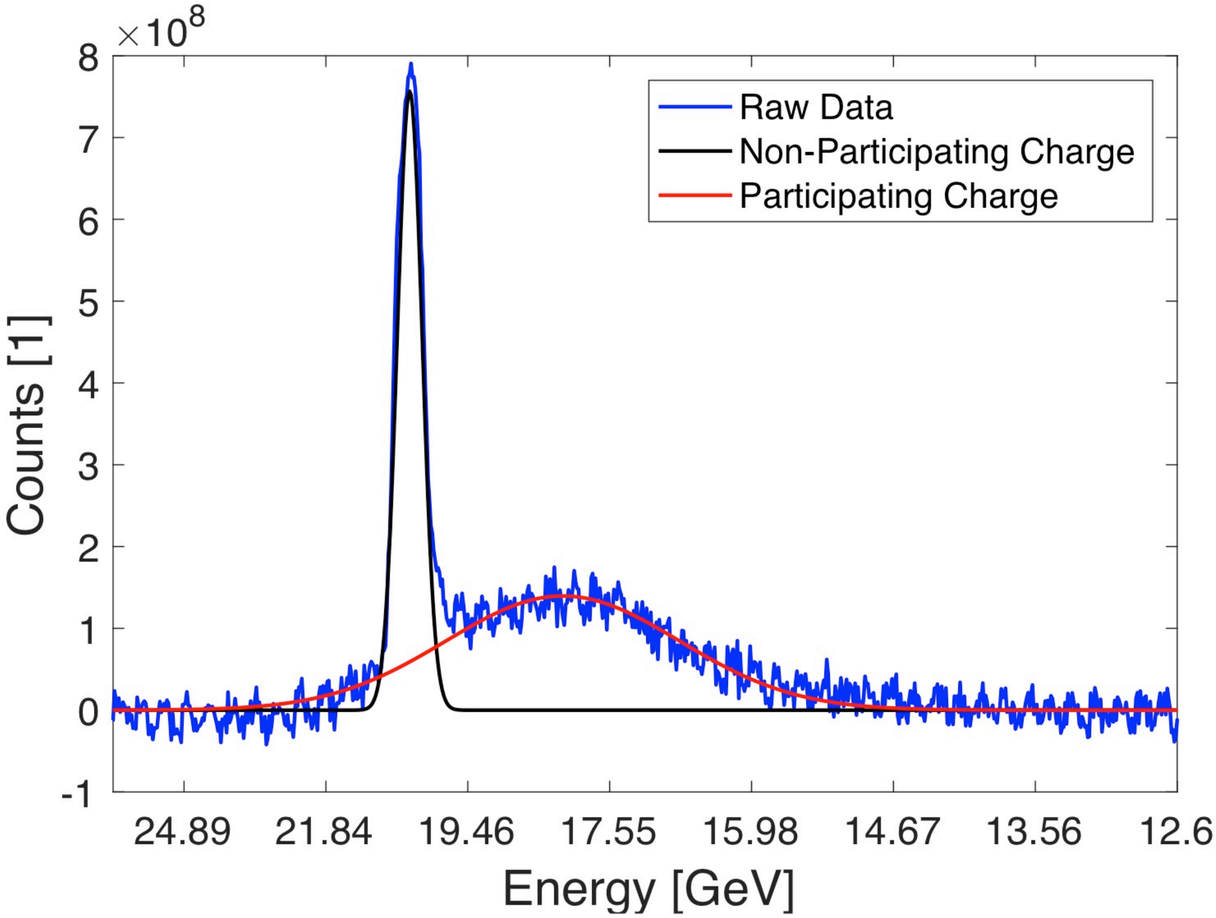

Supplementary Figure 2: Energy spectrum of spent SLAC bunch.

Blue: total spectrum; Black: portion of beam not contributing to wake formation; Red: portion contributing to wake formation. Data recorded by magnetic spectrometer downstream of Li oven.

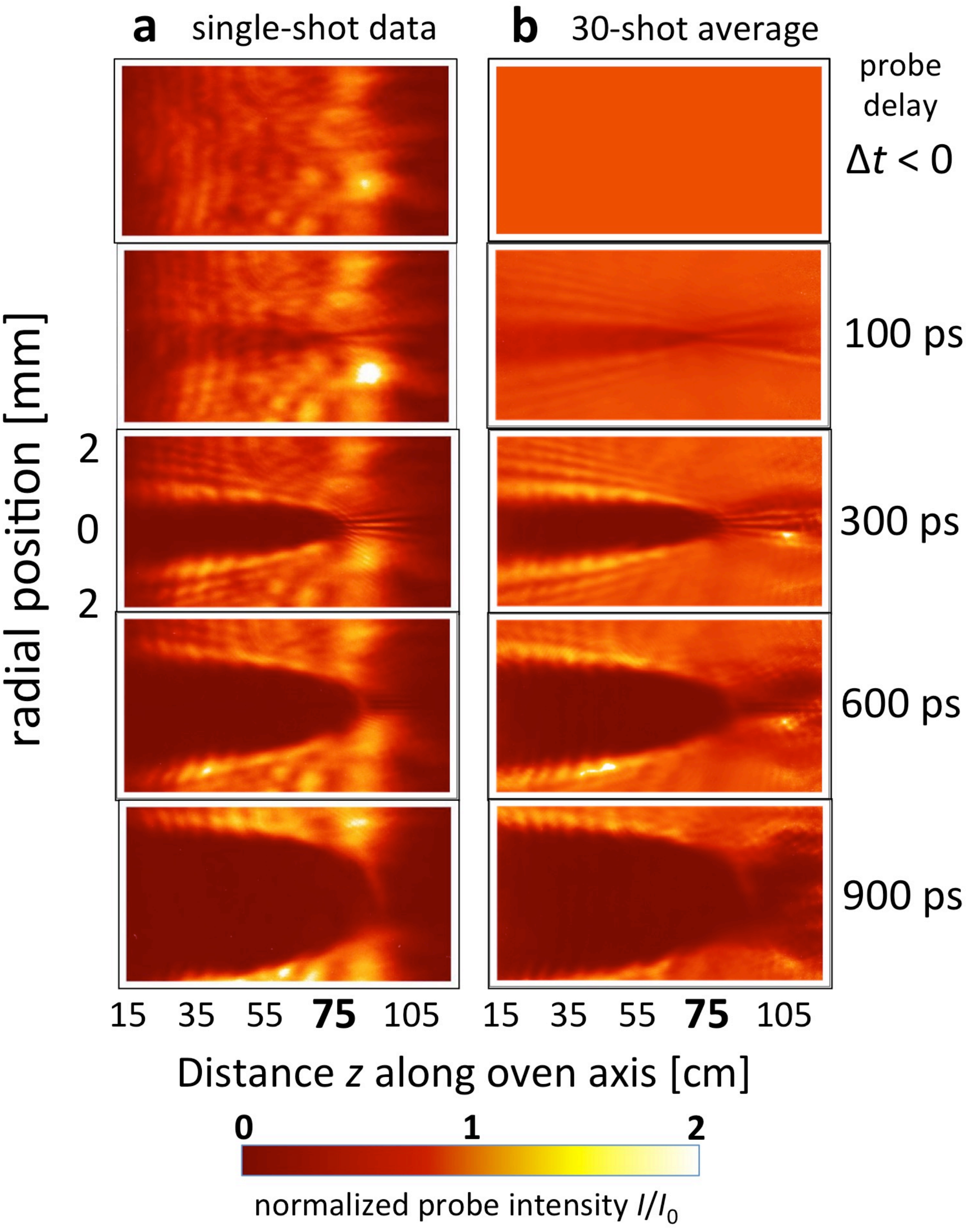

Supplementary Figure 3: Effect of multi-shot averaging on probe data.

a Single-shot and **b** 30-shot-averaged probe images for five different probe delays from the electron drive bunch. Radial position scale shown for 300 ps image applies to all images.

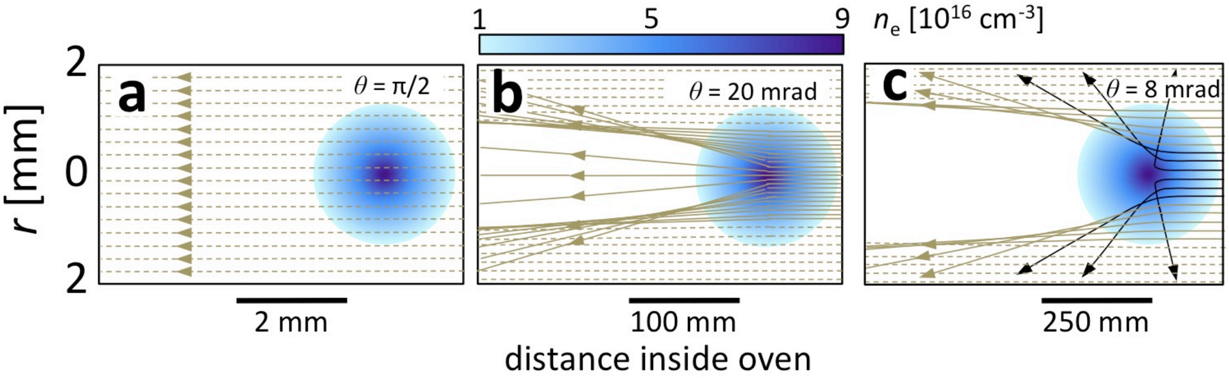

Supplementary Figure 4: Trajectories of probe pulse rays through plasma column.

Cross section of plasma column is shown in blue. Dashed (solid) gold lines are un-deflected (deflected) rays of 0.8 μm wavelength probe. **a** Probe crosses plasma column at angle θ = 90°, resulting in negligible deflection; **b** θ = 20 mrad, maximally deflecting rays that pass through the dense center of the column; **c** θ = 8 mrad, the angle used in experiments. Rays impinging on the center of the column (solid black) are deflected out of a *f*/40 collection cone; those passing through the column's outer edge (solid gold) are deflected within the *f*/40 cone.

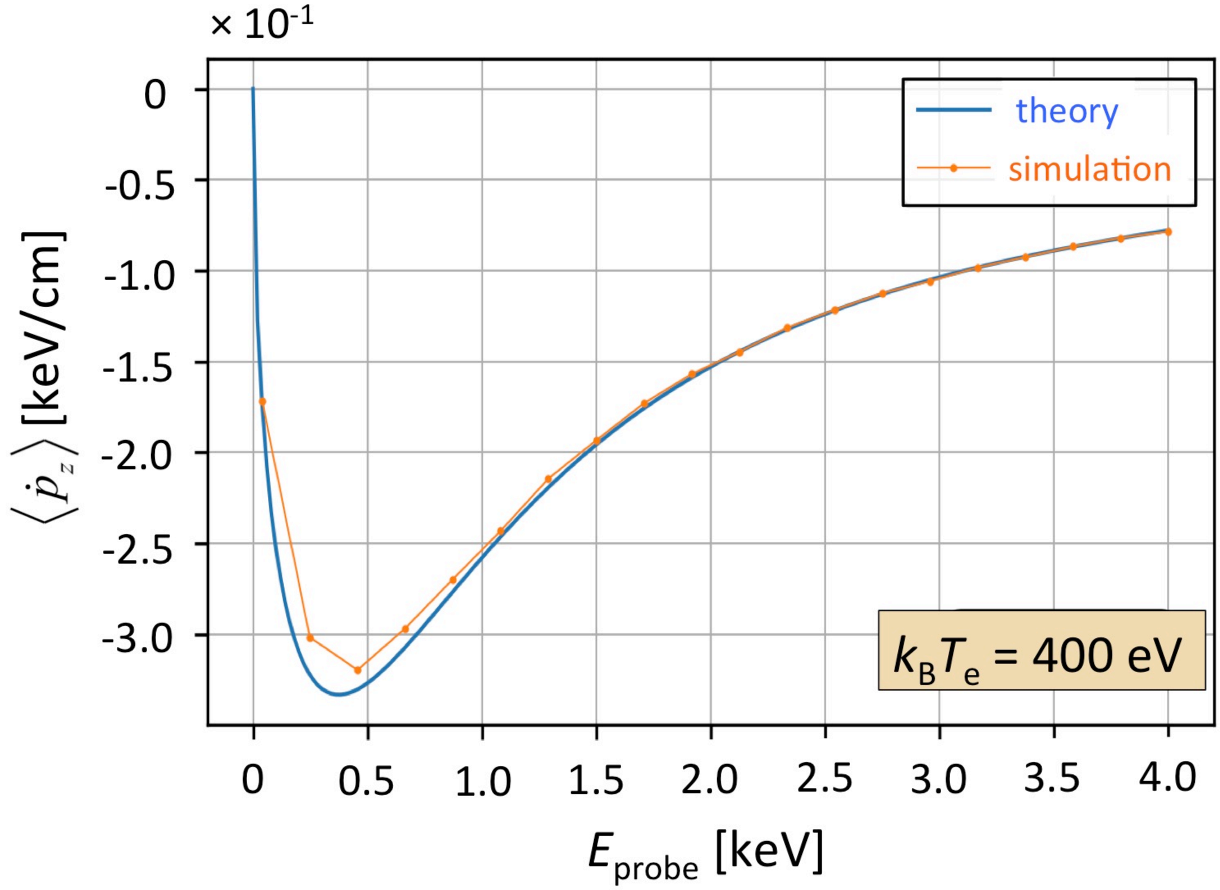

Supplementary Figure 5: Deceleration rate of fast electrons in a medium of Maxwellian electrons.

Orange curve: simulated by LCODE [4] using Takizuka-Abe collision model [5]; blue curve: calculated analytically [10].

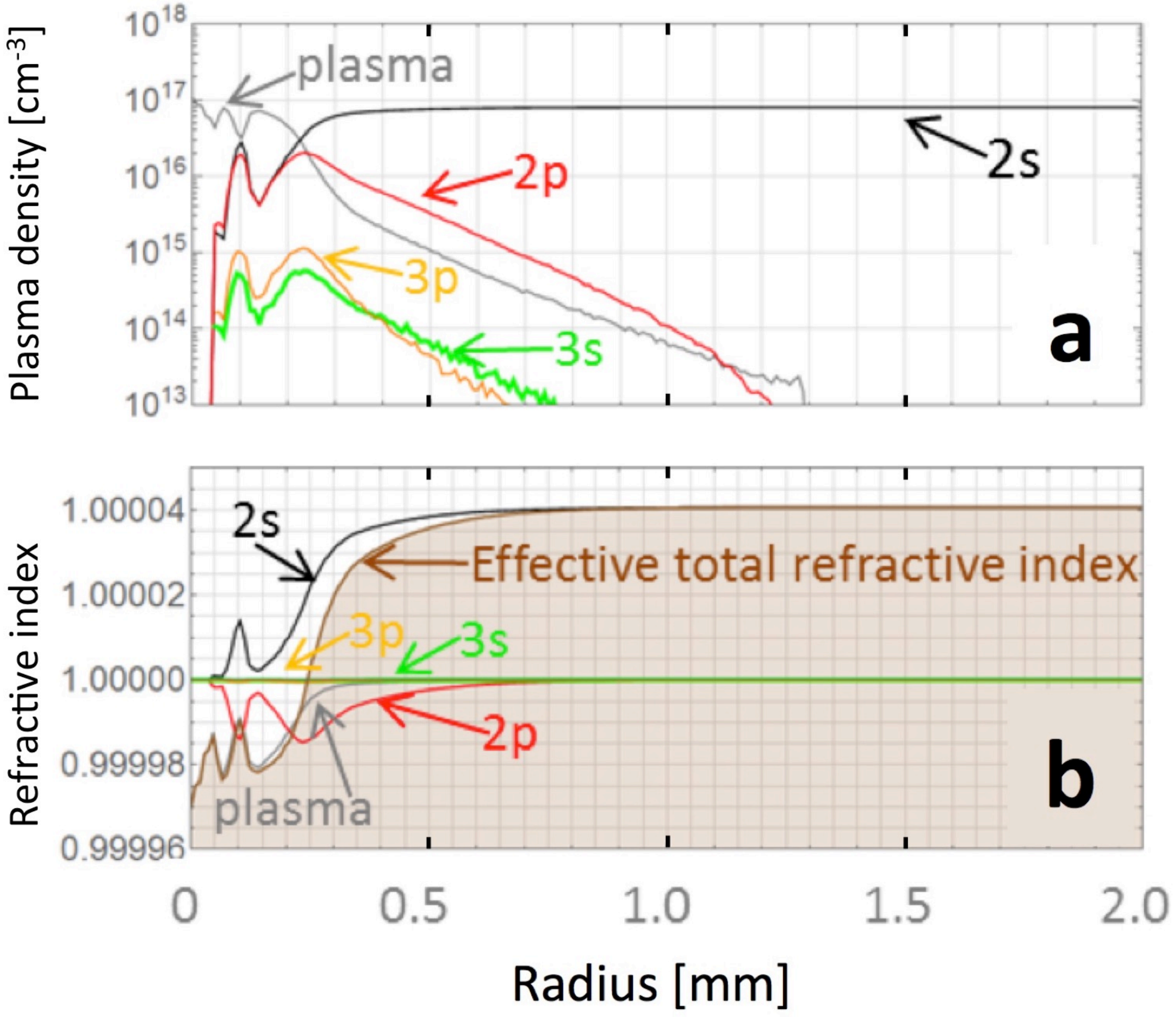

Supplementary Figure 6: Contributions of various Li species to refractive index of e-beam-excited plasma column.

a LCODE result for the radial distribution of five constituent states of lithium at 400 ps after passage of electron beam. **b** Refractive index distributions at 800 nm, corresponding to the densities shown in **a**, including the resultant effective refractive index.

Supplementary Methods

Longitudinal lithium vapor density profile along lithium heat-pipe oven

The Li vapor profile $n_a(z)$ was determined from the temperature profile $T(z)$, measured with a thermocouple probe inserted along the axis of the heat-pipe oven. In the evacuated oven, $T(z)$ is approximately Gaussian with its maximum in the center of the oven ($\Delta z = 0$) and half-maximum at $\Delta z \approx 60$ cm, due to heat conduction along the oven walls. With Li in the oven and the same heater power P_{heat}, $T(z)$ becomes approximately super-Gaussian: nearly flat in the center, and a rapid drop near the ends. This is a consequence of heat conductivity of Li vapor filling the interior and liquid Li spreading along the wick, together with vapor confinement by cold He buffer gas at both ends. Fig. 4 of Muggli *et al.* [1] shows representative $T(z)$ profiles for empty and Li-filled ovens. By modeling the Li-filled oven thermodynamically, and measuring longitudinally integrated density-length product in similar ovens by optical absorption and interferometry [1], we calibrated $T(z)$ in terms of $n_a(z)$. Supplementary Figure 1 shows the calibrated $n_a(z)$ profile used for experiments and simulations described in the main text.

E-beam energy deposition into, and head erosion in, Li plasma

We determined the average rage of energy deposition into the plasma by measuring the energy spectrum of the spent SLAC e-bunch in a magnetic spectrometer located downstream of the Li heat-pipe oven. The spectrum (see Supplementary Figure 2) had two components: (*i*) a sharp peak centered at the incident beam energy (20.35 GeV), consisting of 40% of incident electrons, which evidently did not participate in wake generation (Supplementary Figure 2, black curve); (*ii*) a broadened peak centered at 18.14 GeV, consisting of 60% of incident electrons, which did participate in wake generation (Supplementary Figure 2, red curve). The participating fraction of the beam thus lost (20.35 – 18.14)/20.35 ≈ 0.11 of its energy. This corresponds to 7% of the energy (40 J) of the entire incident beam, or 2.6 J, deposited over 1.2 m, or 2.2 J/m on average.

Since our drive e-beam self-ionized the Li vapor, backward drift of the field-ionization front during propagation gradually erodes the head of the e-beam [2, 3]. Here we estimate the magnitude of this effect for conditions of our experiment. From simulations, the number of μm that the ionization front slips backward per meter of *e*-beam propagation is [2]:

$$V[\mu\text{m/m}] = (3.66 \times 10^4)\,(E_i\,[\text{eV}])^{1.73}\,\gamma^{-1}\,\varepsilon_n\,[\text{mm-mrad}]\,(I\,[\text{kA}])^{-3/2}$$
$$\approx 2.5\ \mu\text{m/m}, \qquad \text{(Supplementary Equation 1)}$$

where we obtained the numerical result using parameters E_i = 5.39 eV (ionization energy of Li), $\gamma = 4 \times 10^4$ (Lorentz factor of 20 GeV drive beam), $\varepsilon_n \approx$ 5 mm-mrad (normalized emittance of FACET-I beam at IP) and $I \approx 10$ kA (peak current of 2 nC, $\sigma_z = 55\ \mu$m FACET-I beam). Since the initial beam length is $\sigma_z = 55\ \mu$m, estimated erosion is < 5% over the 1 m interaction length. This can be considered negligible.

Single-shot vs. 30-shot-averaged probe images

The transverse intensity profile of the probe pulse typically contained non-uniformities, some varying randomly from shot-to-shot, others drifting slowly over minutes to hours. Consequently bright and dark spots that were unrelated to beam-generated plasma structures appeared in recorded single-shot images (see Supplementary Figure 3a). At time delays $\Delta t < 0$, such laser-related artifacts were the *only* features visible in recorded images (see Fig. S3a). A 30-shot average eliminated them (see Supplementary Figure 3b). At $\Delta t > 0$, plasma-induced features appeared over and above laser-profile artifacts in single-shot images (Supplementary Figure 3a), but were more stable over multiple shots. Thus they persisted in 30-shot-averaged images, while artifacts were smoothed out (Supplementary Figure 3b). In Fig. 1c of the main text, we have therefore presented 30-shot averages.

Probe ray tracing for different incidence angles

The deflection angle of a probe ray as it traverses the plasma column, and thus its ability to image the column, depends on the angle θ with which it impinges on the column axis, and on its transverse position within the probe beam profile. Supplementary Figure 4a shows ray trajectories (dashed gold lines) of a transverse ($\theta = \pi/2$) probe traversing a mm-radius cylindrical plasma column (cross section: shaded blue circle) of maximum density $n_e \leq 10^{17}$ cm^{-3}, which approximates a typical plasma column encountered in the reported experiments. The deflection is negligible. Thus a 90° probe could not image such a low-density plasma column, even if this configuration were experimentally accessible. Supplementary Figure 4b shows deflected (solid gold) and negligibly- or un-deflected (dashed gold) ray trajectories of the same probe traversing the same plasma column at angle θ = 20 mrad. The probe ray bundle shown propagates in the plane of the figure, which intersects an elliptical cross section (ellipticity 50) of the plasma column. The horizontal scale has been contracted by a factor of 50, so that the elliptical cross section appears circular (blue). In this geometry, the most strongly deflected rays pass through the dense interior of the plasma column. This geometry would therefore be effective for imaging plasma density structures near the axis. The most strongly deflected rays correspond to an *f*/50 collection cone. Thus the *f*/40 imaging system used in the reported experiments could capture all of them without losing information. This was not done because (a) edges of the heat-pipe oven would have blocked the probe pulse at this angle and (b) our interest was to image the advancing ionization front that defined the plasma column's outer edge.

Supplementary Figure 4c shows corresponding ray trajectories for the actual probe angle θ = 8 mrad used in the reported experiments. Here the intersected elliptical cross section (blue) has ellipticity 125, and the horizontal scale is contracted by the same factor. Now those probe rays that impinge on the plasma column's dense interior deflect at angles outside the *f*/40 collection cone (solid black rays). This information is therefore lost from reported images. Only those

probe rays that pass through the plasma column's outer edge (solid gold) are collected, and contribute to recorded images along with un-deflected rays (dashed gold). The choice of grazing angle θ thus allows one to tune the radial portion of the plasma column to which the images are sensitive.

Test of collisional scheme in LCODE

LCODE [4] treats collisions using well-established collisional/ionization schemes [5-7] that have been successfully implemented and extensively tested in other codes [8, 9], often under far more severe conditions (e.g. relativistic collisions, high density) than exist here. Nevertheless, one effect that is important to modeling plasma column expansion rate in the current experiment --- collisional deceleration of fast (few keV) electrons --- was not fully addressed in available publications. Therefore we developed the following special test of the Takizuka-Abe model [5] for our parameters of interest. A beam of probe electrons with equal momentum (0, 0, p_z) and energy E_{probe} interacts with a medium of Maxwellian electrons with temperature T and density n. The initial deceleration rate is given by the following analytic formula [10]

$$\left\langle \dot{p}_z \right\rangle = -\frac{4\pi n e^4 \lambda}{k_{\text{B}} T} f\left(\frac{E_{\text{probe}}}{k_{\text{B}} T}\right), \qquad \text{(Supplementary Equation 2)}$$

where $f(x) = \frac{erf(\sqrt{x})}{x} - \frac{2}{\sqrt{\pi x}} e^{-x}$, k_{B} is Boltzmann's constant and λ is the Coulomb logarithm.

For simulations, medium and probe electrons were distributed among 500 cells, each with 125 ppc (total 62500 particles) of each species. We performed collisions using a small time step dt (typically 1/5000 of the expected deceleration time) and followed the momentum p_z(t) of probe particles for 50 steps. After each time step, the distribution of medium particles is restored to its initial Maxwellian distribution, as assumed in the theoretical formula. The result is averaged over 62500 probe particles. Comparison between theory and simulation for different

probe energies is given in Supplementary Figure 5. The high-E_{probe} part of curve p_z(t) gives us the deceleration rate of interest, demonstrating accurate correspondence between simulation and theory.

Atomic, ionic and plasma contributions to Li refractive index

Here we exhibit contributions of various Li species to the calculated refractive index of the e-beam-excited plasma column. Supplementary Figure 6a shows an example of simulated radial density distribution of five constituent states/species considered --- neutral lithium in the 2s, 2p, 3s and 3p states, and singly ionized lithium --- at Δt = 400ps. Supplementary Figure 6b shows their corresponding contributions to the refractive index. The brown curve and shaded area show the overall effective refractive index. Methods/Simulations [Equations (1) to (3), and accompanying text] describes how these curves were calculated. These results form the basis of the families of plots shown in Fig. 4 of the main text.

The effective refractive index depends on partial fractions of the five states/species, which vary with time and radius, leading to a complex time-varying evolution of the medium's refractive index. In most cases plasma refractive index η_5 [Equation (2) of main text] dominates in the core of the plasma column, while the neutral 2S state refractive index η_1 [Equation (1) of main text] dominates at larger radii. The transition between these evolves with time. The relative importance of the 2p, 3s, and 3p states is lower, but becomes significant at radii near the transition from ionized to partly ionized lithium.

Supplementary References

1. Muggli, P., Marsh, K. A., Wang, S., Clayton, C. E., Lee, S., Katsouleas, T. C. and Joshi, C. Photo-ionized lithium source for plasma accelerator applications. *IEEE Trans. Plasma Science* **27**, 791-799 (1999).

2. Li, S. Z., Adli, E., England, R. J., Frederico, J., Gessner, S. J., Hogan, M. J., Litos, M. D., Walz, D. R., Muggli, P., An, W. and Clayton, C. E. Head erosion and emittance growth in PWFA. *Proceedings 2012 Advanced Accelerator Concepts Workshop*, https://aip.scitation.org/doi/abs/10.1063/1.4773762 (2012).

3. Vafaei-Najafabadi, N., Marsh, K. A., Clayton, C. E., An, W., Mori, W. B., Joshi, C., Lu, W., Adli, E., Corde, S., Clarke, C. I. and Litos, M. Limitation on accelerating gradient of a wakefield excited by an ultrarelativistic electron beam in rubidium plasma. *Phys. Rev. Accel. Beams* 19, 101303 (2016).

4. Sosedkin, A. and Lotov, K. LCODE: A parallel quasistatic code for computationally heavy problems of plasma wakefield acceleration. *Nucl. Instum. Meth. Phys. Res. A* **829**, 350-352 (2016).

5. Takizuka, T. and Abe, H. A binary collision model for plasma simulation with a particle code. *J. Comp. Phys.* **25**, 205 (1977).

6. Younger, S. Electron impact ionization of lithium. *J. Res. Natl. Bur. Standards* **87**, 49-51 (1982).

7. Kaganovich, I. D., Startsev, E. and Davidson, R. C. Scaling and formulary of cross-sections for ion-atom impact ionization. *New J. Phys.* **8**, 278 (2006).

8. Pérez, F., Gremillet, L., Decoster, A., Drouin, M. and Lefebvre, E. Improved modeling of relativistic collisions and collisional ionization in particle-in-cell codes. *Phys. Plasmas* **19**, 083104 (2012).

9. Arber, T. D., Bennett, K., Brady, C. S., Lawrence-Douglas, A., Ramsay, M. G., Sircombe, N. J., Gillies, P., Evans, R. G., Schmitz, H., Bell, A. R. and Ridgers, C. P. Contemporary particle-in-cell approach to laser-plasma modeling. *Plasma Phys. Control. Fusion* **57**, 113001 (2015).

10. Trubnikov, B. A., Particle interactions in fully ionized plasma. *Rev. Plasma Phys*. **1**, 105-140 (1965).
